# Multicomponent exercise training in cardiovascular complexity in prefrail older adults: a randomized blinded clinical pilot study

**DOI:** 10.1590/1414-431X202010794

**Published:** 2021-04-26

**Authors:** M.S.S. Buto, V. Vassimon-Barroso, E. Fiogbé, A.C.S. Farche, B.F. Carnavale, P.G. Rossi, C.A. Sakaguchi, A.M. Catai, A.C.M. Takahashi

**Affiliations:** 1 Departamento de Fisioterapia, Universidade Federal de São Carlos, São Carlos, SP, Brasil

**Keywords:** Aging, Baroreflex, Complexity, Frailty, Physical exercise

## Abstract

The aim of this study was to investigate the effects of multicomponent training on baroreflex sensitivity (BRS) and heart rate (HR) complexity of prefrail older adults. Twenty-one prefrail community-dwelling older adults were randomized and divided into multicomponent training intervention group (MulTI) and control group (CG). MulTI performed multicomponent exercise training over 16 weeks and CG was oriented to follow their own daily activities. The RR interval (RRi) and blood pressure (BP) series were recorded for 15 min in supine and 15 min in orthostatic positions, and calculation of BRS (phase, coherence, and gain) and HR complexity (sample entropy) were performed. A linear mixed model was applied for group, assessments, and their interaction effects in supine position. The same test was used to assess the active postural maneuver and it was applied separately to each group considering assessments (baseline and post-intervention) and positions (supine and orthostatic). The significance level established was 5%. Cardiovascular control was impaired in prefrail older adults in supine position. Significant interactions were not observed between groups or assessments in terms of cardiovascular parameters. A 16-week multicomponent exercise training did not improve HR complexity or BRS in supine rest or in active postural maneuver in prefrail older adults.

## Introduction

There is accumulating evidence that frailty may become one of the world's most serious health issues (1). Considering the expansive increase of the older adult population in the world, frailty prevalence tends to rise considerably (2) and consequently a burden on health and elderly care systems are also expected (1,3).

In this context, frailty appears as one of the most problematic conditions, described as a clinical state of vulnerability to stress as a consequence of the decline of resilience and physiologic reserve related to aging, resulting in increased risk of adverse outcomes such as mortality, falls, institutionalization, hospitalization, loss of independence, and progressive decline in homeostasis (3–5).

The maintenance of homeostasis depends on a complex network of interactions among the control mechanisms. The aging process is accompanied by a reduction of these interactions in the physiological systems, which limits adequate response to stressors and characterizes the organism with reduced physiological complexity (6,7). In frailty, a more pronounced loss of physiological complexity occurs, which would induce a loss of functional capacity to critical levels. Thus, the individual would become less resilient and therefore more vulnerable to development of pathologies and adverse outcomes as mentioned above (7).

Currently, the study of physiological complexity has been suggested in addition to traditional measurements in biological and health research (8). Once the physiological systems present a dynamic behavior, the complexity approach may offer an opportunity to characterize qualitatively these interactions (7), as well as baroreflex sensitivity (BRS) may represent interactions for blood pressure (BP) control. In this sense, one of the physiological systems most studied is the cardiovascular system (7,9). It has already been demonstrated that frail older adults present impairment in cardiac complexity (5,10,11) and in BRS (12).

Among the several interventions designed for frail and prefrail older adults, multicomponent exercise training has been demonstrated as the most effective for the reverse of frailty status and benefits in physical domains (13,14). Nonetheless, the underlying physiological mechanisms remain unclear. To date there is little and divergent information about reversibility of cardiovascular complexity. Resistance training is effective for heart rate (HR) complexity improvement in young individuals as well as in hypertensive older adults (15,16), but contrarily, it does not improve BRS in middle-aged people (17).

Thus, it remains unknown if an exercise intervention could promote benefits in cardiovascular complexity in prefrail elderly. In this sense, the aim of the present study was to verify if multicomponent exercise training could restore HR complexity as well as BRS in prefrail older adults.

## Material and Methods

### Ethical approval

This blinded randomized controlled trial was registered (Clinical Trial Registration ID: NCT03110419) and approved by the Research Ethics Committee of the institution (ID: 1800231/2016). Written consent was obtained from all the volunteers. All procedures were performed in accordance with the ethical standards of the 1964 Helsinki Declaration.

### Sample

The inclusion criteria were community-dwelling prefrail older adults (according to frailty phenotype (4)) ≥65 years old, with medical approval for exercising, and who agreed to participate in the study. The non-inclusion criteria were: a) Parkinson's disease; b) stroke; c) diabetes mellitus with peripheral neuropathy (18) assessed by Semmes-Weinstein monofilaments at 5.07 (10 g) (19); d) vestibular and visual self-reported disorders that would impair performance in assessment and/or training; e) an indication of cognitive deficit, assessed by means of the Mini Mental State Examination (MMSE) with scores lower than 18 (4); f) cardiovascular alterations (atrial fibrillation, malignant ventricular arrhythmia, complex ectopic ventricular beat, sinus or supraventricular tachycardia, second and third degree atrioventricular block; g) use of a pacemaker on resting electrocardiogram (ECG); h) unstable angina; and i) myocardial infarction.

**Figure 1:**
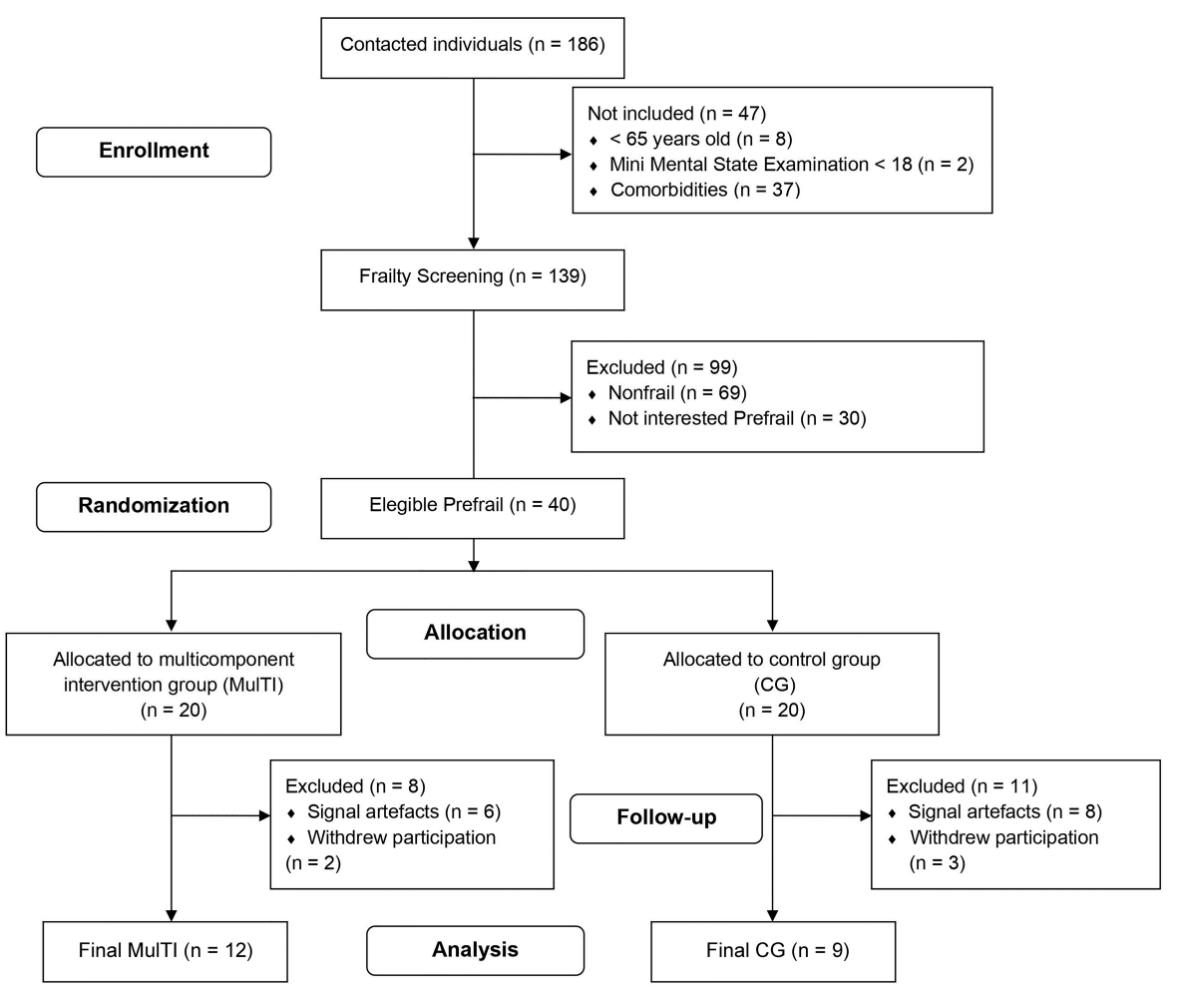
Flowchart of the final sample.

Figure 1 shows a flowchart of the sample. Initially, 186 older adults were contacted. Forty-seven were not included due to age, comorbidities according to criteria, and MMSE score <18. Frailty screening was applied in 139 individuals and 99 were excluded due frailty status or non-interest in the study. Finally, 40 were considered eligible and were randomized into two groups of 20 subjects: i) the multicomponent training intervention group (MulTI), which participated in a multicomponent physical exercise protocol and ii) the control group (CG), which was oriented to follow their own habitual daily activities. After participation withdrawals and removal of signal artefacts, the final sample was composed by 21 subjects: MulTI (n=12) and CG (n=9) (see Figure 1).

### Study protocol

#### Randomization process

After the first assessment, the participants were randomly distributed using the Random Allocation Software (Microsoft Corporation, USA) into blocks of eight subjects. According to the randomization sequence, each participant was allocated according to a numbered card sealed in an opaque envelope indicating which group the individual would be inserted (MulTI or CG). The entire randomization process was performed by a researcher who had no link to the study (J.H.A.). The envelopes were opened after the first evaluation and the researchers were blinded about the allocation of the participants.

The assessments were performed at two distinct times: 1) baseline (initial assessment) and post-intervention (immediately after the conclusion of the 16-week multicomponent intervention).

#### Anamnesis

All participants were submitted to a structured interview. Demographic data (age and sex), educational level, MMSE total score, Short Physical Performance Battery (SPPB) total score, and comorbidities data were collected.

#### Procedures and experimental protocol

Initially, the volunteers were instructed to remain in supine position for 10 min in order to stabilize cardiovascular variables and to conduct the calibration procedure. ECG, blood pressure (BP), and breathing recordings were collected for 15 min in supine position. Then, the volunteers were instructed to actively change to orthostatic position, in which they remained for 15 min. Previous instructions were given related to not ingest caffeine or alcohol or perform moderate or heavy exercise on the day before participation and to have a regular meal.

The experiments were conducted in a climate-controlled (22-23°C) room with relative air humidity of 40-60% always in the morning in order to minimize circadian cycle effects.

#### Signal acquisition

The ECG signal was collected by a bioamplifier (BioAmp Power Lab, Ad Instruments, Australia) with electrodes placed on the MC5 lead, and respiratory movements were captured by a respiratory belt (Marazza, Italy). The arterial BP waves were obtained by a plethysmography arterial pressure device (Finometer PRO, Finapress Medical Systems, The Netherlands), with a cuff placed on the distal extremity of the right middle finger. The right hand was kept close to the volunteer's heart with the help of a sling, which fixed the volunteer's arm to his chest throughout the experiment. The signal acquisition frequency was sampled at 1000 Hz.

The extraction of beat-to-beat variability series was carried out according to previous descriptions (20). After extraction of the series, the 256-point sequences with the greatest stability were chosen for both positions (21).

### Baroreflex sensitivity

Baroreflex was evaluated by phase, coherence (K^2^), and gain (α). Baroreflex was calculated by cross-spectral analysis using a bivariate autoregressive model (22). The phase is computed as the phase of the cross-spectrum from BP to RR interval (RRi) and represents the delay between the change in BP and the subsequent change in RRi, measured in radians. Coherence (K^2^) was used to estimate the strength of the coupling between RRi and BP. The squared coherence is computed as the ratio of the squared modulus of the cross-spectrum to the product of the power spectra. In this study, phase and coherence were sampled at the frequency of vasomotor oscillations (Mayer waves) at the low frequency band (LF), which oscillates between 0.04-0.15 Hz and is related to the sympathetic predominance (21,23). Gain in the LF band was calculated as the square root of the ratio of the LF power of the RRi series to that of the BP series (24) and characterizes the relation between BP and RRi.

### Sample entropy (SampEn)

Entropy is a measure of the information needed to predict the future state of a system. It provides a characterization of the dynamics of a signal, the greater the dynamics, the greater the entropy and the less predictable the system (6,7).

Sample entropy (SampEn) (25) is a complexity measure used to quantify regularity of time series, especially short and noisy sequences. It is a measure that monitors how much a set of patterns are close together for a few observations. Lower values for SampEn indicate regularity and predictability. In this study, it was computed with m=2, r=0.2 times the standard deviation of the signal, and n=256. M represents the length of the vector (patterns) to be compared and r represents the radius within which the comparison between the vectors is achieved (similarity criteria).

### Intervention protocol

The multicomponent exercise intervention was designed considering the recommendations proposed by the American College of Sports Medicine (26). The protocol consisted of aerobic, muscle strength, flexibility, and balance exercises. It was performed during 16 weeks, for three non-consecutive days, with 60-minute sessions. The sessions were composed by: a) 10 min of warm-up (light walk); b) 20 min of aerobic exercises; c) 10 min of balance exercise; d) 15 min of resistance exercises; and e) 5 min of cool-down. Details concerning exercise type, intensity, and progression are described in a previous study (27).

Before the beginning of the intervention, all volunteers of the MulTI were invited to participate in three sessions on non-consecutive days for familiarization process and determination of resistance training load.

### Statistical analysis

Sample size calculation was based on a previous study (11) that presented cardiovascular parameters as main outcome in prefrail and frail population. The Shapiro-Wilk test was used to verify the normality of data distribution. The Student's paired *t*-test was used to compare age, anthropometric characteristics (weight, stature, BMI), number of comorbidities, educational level, MMSE total score, and SPPB total score at baseline. The chi-squared test was applied to compare sex and each frailty criteria according to phenotype.

A linear mixed model test was used to assess the effect of the training at baseline and post-intervention, between MulTI and CG on functional measures (walk distance and gait speed) and on cardiovascular variables in supine rest position. The same test was used to assess the effect on postural maneuver separately for each group, considering assessments (baseline × post-intervention) and positions (supine × orthostatic) in cardiovascular variables.

The significance level established was 5%. Statistical analysis was performed using the software IBM SPSS Statistics, version 20.0 (USA).

## Results

The volunteers' age, and anthropometric and clinical characteristics are presented in Table 1. There was no difference in sex, age, weight, stature, number of comorbidities, educational level, MMSE total score, and frailty criteria between the groups. There was significant difference for BMI (P=0.032) and SPPB total score (P=0.006) between the groups.

**Table 1:** Age, anthropometric, and clinical characteristics.

	MulTI (n=12)	CG (n=9)	P-value
Female gender, n (%)	10 (83.3)	5 (55.6)	0.163
Age (years)	77.00±6.80	73.78±6.28	0.281
Weight (kg)	76.26±12.72	68.54±8.69	0.135
Stature (cm)	154.83±6.48	159.56±5.90	0.102
BMI (kg/m^2^)	31.95±5.47	27.02±3.74	**0.032**
Comorbidities	2.33±1.30	2.44±1.81	0.872
Educational level (years)	4.42±2.57	6.83±4.37	0.207
MMSE total score	24.50±2.54	25.89±3.95	0.339
SPPB total score	7.58±1.31	9.44±1.42	**0.006**
Frailty criteria			
Unintentional weight loss, n (%)	3 (25.00)	2 (22.22)	0.647
Low activity level, n (%)	3 (25.00)	3 (33.33)	0.523
Weakness, n (%)	3 (25.00)	2 (22.22)	0.647
Exhaustion, n (%)	6 (50.00)	4 (44.44)	0.575
Slowness, n (%)	2 (16.66)	0 (0.00)	0.314

Data are reported as means±SD or total of individuals (percent). MulTI: multicomponent training intervention; CG: control group; BMI: body mass index; MMSE: Mini Mental State Examination; SPPB: Short Physical Performance Battery. P<0.05 compared to CG (Student’s paired *t-*test and chi-squared test).

Table 2 presents BRS and SampEn results in rest supine position. In terms of BRS, there was no difference in any parameter. Concerning SampEn, despite groups, there was a significant reduction in post-assessment compared to baseline (P=0.036).

**Table 2:** SampEn and baroreflex sensitivity in MulTI and CG groups in supine position.

	MulTI		CG		P-value		
	Baseline	Post	Baseline	Post	Groups	Assessments	Interaction
SampEn	1.72±0.58	1.30±0.46	1.72±0.38	1.32±0.43	0.971	0.036*	0.945
BRS							
Coherence	0.51±0.12	0.36±0.16	0.51±0.17	0.55±0.28	0.992	0.076	0.117
Phase (rad)	-1.10±1.19	-1.76±1.09	-1.16±1.61	-0.81±2.59	0.830	0.573	0.889
Gain (ms/mmHg)	5.03±3.59	7.07±4.00	3.47±1.45	7.43±5.84	0.521	0.239	0.727

Data are reported as means±SD. SampEn: Sample entropy; BRS: baroreflex sensitivity; MulTI: multicomponent training intervention; CG: control group. *P<0.05 compared to baseline (baseline > post, linear mixed model test).

The postural maneuver behavior of each group between assessments is reported in Tables 3 and 4. Thus, the effects of positions (supine × orthostatic) and assessments (baseline × post-intervention) were tested for each group considering BRS and SampEn. The CG did not present significant differences for position, assessment, or interaction in BRS parameters. Nonetheless, there was a reduction in post-intervention assessment related to baseline for SampEn (P=0.008) (Table 3). The MulTI group did not present any significant differences for BRS or SampEn values between position, assessments, or their interaction (Table 4).

**Table 3:** SampEn and baroreflex sensitivity (BRS) in control group.

	Baseline		Post		P-value		
	Supine	Orthostatic	Supine	Orthostatic	Position	Assessments	Interaction
SampEn	1.72±0.38	1.75±0.50	1.32±0.43	1.06±0.33	0.880	0.008*	0.366
BRS							
Coherence	0.51±0.17	0.50±0.22	0.55±0.28	0.58±0.23	0.942	0.314	0.719
Phase (rad)	-1.16±1.61	-1.77±0.84	-0.81±2.59	-1.34±0.47	0.410	0.615	0.966
Gain (ms/mmHg)	3.47±1.45	4.14±2.70	7.43±5.84	4.23±2.99	0.684	0.932	0.082

Data are reported as means±SD. SampEn: sample entropy. *P<0.05 compared to baseline (baseline > post, linear mixed model test).

**Table 4:** SampEn and baroreflex sensitivity (BRS) in multicomponent training intervention group.

	Baseline		Post		P-value		
	Supine	Orthostatic	Supine	Orthostatic	Position	Assessments	Interaction
SampEn	1.72±0.58	1.53±0.43	1.30±0.46	1.36±0.42	0.320	0.231	0.316
BRS							
Coherence	0.51±0.12	0.49±0.13	0.36±0.16	0.46±0.19	0.730	0.593	0.152
Phase (rad)	-1.10±1.19	-1.87±0.89	-1.76±1.09	-1.49±1.34	0.106	0.385	0.090
Gain (ms/mmHg)	5.03±3.59	2.76±1.99	7.07±4.00	4.01±4.65	0.133	0.449	0.713

Data are reported as means±SD. SampEn: sample entropy. Linear mixed model test.

Considering the functional measures, there was a group effect for walk distance (P=0.019) indicating the CG presented higher values compared to MulTI. Furthermore, CG also presented higher values for gait speed than MulTI (P<0.001) and in post-intervention assessment both groups presented a better performance (increase) compared to baseline (P=0.001) (Table 5).

**Table 5:** Functional measures of the multicomponent training intervention group (MulTI) and control group (CG).

Functional measures	MulTI		CG		P-value		
	Baseline	Post	Baseline	Post	Groups	Assessments	Interaction
Walk distance (6MWT) (m)	326.42±64.42	323.50±83.41	401.00±53.18	414.00±58.42	0.019*	0.741	0.375
Gait speed (m/s)	0.74±0.20	0.91±0.16	1.05±0.12	1.11±0.14	<0.001*	0.001*	0.183

Data are reported as means±SD. 6MWT: six-minute walk test. *P<0.05 compared to baseline (post > baseline, linear mixed model test) and between groups (MulTI *vs* CG, linear mixed model test).

## Discussion

Complexity is an elusive concept, which has been inserted in biological and health studies once physiological systems are featured by a dynamic network of multiple interacting inputs between control mechanisms (7). Physiological complexity is directly related to an adaptive capacity of the organism to ever-changing environment (28). Thus, a healthy organism is characterized by presence of adaptability properties, which allows effective coping and high functionality to respond to unpredictable stimuli and stresses of daily life (7,29).

With the aging process, the number and connectedness of these inputs is reduced and the output signal is simplified, which limits responses to stressors and features as a reduced physiological complexity (7). As complexity falls further, it may impair functional capacity until crossing the frailty threshold (7), resulting in evident vulnerability to adverse outcomes (4). Therefore, the greater the number of dysregulated physiological systems, the stronger the likelihood of frailty development (30).

Previous studies identified impairment in cardiovascular control assessed by complexity measurements in frail older adults (7,10,11). On the other hand, it is unclear if this impairment could be present in prefrail older adults, even in a lesser proportion. Structural and functional alterations of noninvasive biomarkers of cardiovascular disease (CVD) such as level of carotid stenosis and wall thickness are prevalent in frail as well as in prefrail individuals (31). Also, the negative influence of CVD in HR complexity has already been demonstrated (32). According to our findings, HR complexity is impaired in prefrail older adults even in rest supine condition, once a significant reduction was detected in the post-assessment compared to baseline, despite the group.

Once the network structure of the physiological system enables alternate pathways to be used to achieve the same functions, even in adverse conditions as aging or disease, the organism may keep functional capacity if other neural components and their connections could compensate (7). Nevertheless, in frailty course probably there is a limited response repertoire due to lesser interaction among the physiological systems; consequently, the individual may present a too succinct/insufficient or exacerbated response. An example of this is the orthostatic hypotension described in frail individuals (33). Considering that the baroreflex represents an interaction among control subsystems responsible for BP homeostasis (34), in agreement with a previous study (12), our data suggested there is also an impairment in BRS in prefrail individuals.

The active postural maneuver is a functional task that triggers some physiological alterations in cardiac contractility, vasoconstriction, and HR by increasing in sympathetic modulation and vagal withdrawn (35,36). Thus, it is expected that the baroreflex mechanism acts by a fast increase in HR and BP dropping until it restores to adequate levels. Concerning the cardiovascular dynamics response to postural maneuver, a healthy organism presents a decrease in gain of BRS (35,37), increase in K^2^ values (37), as well a decrease in HR complexity (9). In the frailty course, it seems the mechanisms fail and the response is impaired, as shown by the performance of prefrail individuals in the present study once both groups did not respond adequately.

It remains unknown if complexity of cardiovascular control could be restored by any kind of intervention. To date, few studies have been developed aiming to investigate the effect of exercise intervention on cardiovascular complexity and BRS. Resistance training conducted among hypertensive older adults and young individuals promoted an increase in HR complexity, whereas no change was identified in traditional measurements of HR (15,16). Concerning endurance training, it was demonstrated that a 21-week progressive program was more effective in improvement (increase) of HR complexity in middle-aged women compared to combined strength and endurance training or strength training alone (38). Similarly, 4-week endurance training in hypertensive middle-aged individuals improved BRS while the strength training had the opposite effect (17).

The cardiovascular benefits from distinct exercise training are divergent and the underlying mechanisms are still uncertain. It has been suggested that physical exercise, especially endurance and resistance training, could potentially restore at least partially the complex dynamics in physiological systems (5) through the development of new network connections as well as a reorganization of information outflow (5,7) and, consequently, improve functional health.

Currently, there are few intervention studies destined to physiological complexity outcomes. It has been assumed that a protocol design that targets multi-systems effects and treats risk factors of disability may have the greatest potential to restore healthy dynamics in biological systems (5). In consonance, multicomponent exercise training has been considered as the most adequate intervention modality to frailty management especially in earlier stages (13,14).

In this sense, it was hypothesized that the multicomponent intervention protocol developed in the present study would be capable to improve the dynamics evolved in cardiovascular control. On the assumption that frailty is featured by multisystem dysregulation (30), it was thought a broad approach could mutually affect multiple physiological “gears” and restore their interaction, reflecting in HR complexity as well BRS. Nonetheless, our findings indicated this intervention did not improve these parameters. It is possible the protocol design (load, duration, and/or progression) was not the most adequate.

Some authors argue that although an intuitive rationality guides multicomponent approaches, the complex systems theory suggests the modification of a single component of a system may contribute to global (holistic) effects on system behavior (39). This seems to be consistent with theoretical basis that considers the frailty progression as dependent at least to one abnormal system to be able to trigger a downward spiraling and affect other healthy functional systems until achieving a whole dysregulated state (30).

Related to the functional measures, there was no effect of multicomponent training in walk distance and gait speed in prefrail individuals. At baseline and even post-intervention, CG presented better performance (greater walk distance and faster gait speed) compared to MulTI. The aerobic load developed in the multicomponent intervention (60 min/week) might not be enough to promote benefits. According to the American College of Sports Medicine (26), at least 150 min/week of moderate-intensity aerobic exercise has been suggested for older adults.

Lastly, gait speed has been considered as a prognostic predictive factor for all-cause mortality in older patients with cardiovascular disease (40), and also complexity indexes have already proven their prognostic value in some pathological conditions as coronary artery disease (32). Thus, usage of metrics to quantify complexity in addition to functional measurements may contribute to the recommendations designated to specific programs for prefrail individuals and be a potential method of risk stratification for this population (8).

### Conclusions

Prefrail individuals demonstrated a reduction in HR complexity in rest condition, which confirms impairment in the autonomic nervous system related to cardiovascular control even in intermediate frailty stage. Concerning postural maneuver, they did not present the expected response, suggesting a difficulty to deal with provocative tasks that affect homeostasis. Furthermore, the 16-week multicomponent exercise training did not improve HR complexity, BRS, and functional measures (walk distance and gait speed).

Frailty management is a challenge because it presents specificities related to wide and multidirectional physiological features. In this sense, the earliest identification of systemic deficits through sensitive tools may help in the development of effective interventions targeted to prefrail individuals. Future studies should be conducted testing the efficacy of different types of training on cardiovascular dynamics and also on functional measures in a large sample.

### Study limitations

The cardiopulmonary exercise testing, which is considered the gold standard tool for determination of maximal oxygen uptake (VO_2_ max), was not performed to assess aerobic capacity.
